# Machine Learning-Based Radiomics Nomogram With Dynamic Contrast-Enhanced MRI of the Osteosarcoma for Evaluation of Efficacy of Neoadjuvant Chemotherapy

**DOI:** 10.3389/fonc.2021.758921

**Published:** 2021-11-15

**Authors:** Lu Zhang, Yinghui Ge, Qiuru Gao, Fei Zhao, Tianming Cheng, Hailiang Li, Yuwei Xia

**Affiliations:** ^1^ Department of Medical Imaging, People's Hospital of Zhengzhou University Henan Provincial People's Hospital, Zhengzhou, China; ^2^ Department of Orthopedics, People's Hospital of Zhengzhou University Henan Provincial People's Hospital, Zhengzhou, China; ^3^ Department of Radiology, Henan Provincial Cancer Hospital, Zhengzhou, China; ^4^ Huiying Medical Technology Co., Ltd., Beijing, China

**Keywords:** osteosarcoma, MRI, radiomics, nomogram, neoadjuvant chemotherapy

## Abstract

**Objectives:**

This study aims to evaluate the value of machine learning-based dynamic contrast-enhanced MRI (DCE-MRI) radiomics nomogram in prediction treatment response of neoadjuvant chemotherapy (NAC) in patients with osteosarcoma.

**Methods:**

A total of 102 patients with osteosarcoma and who underwent NAC were enrolled in this study. All patients received a DCE-MRI scan before NAC. The Response Evaluation Criteria in Solid Tumors was used as the standard to evaluate the NAC response with complete remission and partial remission in the effective group, stable disease, and progressive disease in the ineffective group. The following semi-quantitative parameters of DCE-MRI were calculated: early dynamic enhancement wash-in slope (Slope), time to peak (TTP), and enhancement rate (R). The acquired data is randomly divided into 70% for training and 30% for testing. Variance threshold, univariate feature selection, and least absolute shrinkage and selection operator were used to select the optimal features. Three classifiers (K-nearest neighbor, KNN; support vector machine, SVM; and logistic regression, LR) were implemented for model establishment. The performance of different classifiers and conventional semi-quantitative parameters was evaluated by confusion matrix and receiver operating characteristic curves. Furthermore, clinically relevant risk factors including age, tumor size and site, pathological fracture, and surgical staging were collected to evaluate their predictive values for the efficacy of NAC. The selected clinical features and imaging features were combined to establish the model and the nomogram, and then the predictive efficacy was evaluated.

**Results:**

The clinical relevance risk factor analysis demonstrates that only surgical stage was an independent predictor of NAC. A total of seven radiomic features were selected, and three machine learning models (KNN, SVM, and LR) were established based on such features. The prediction accuracy (ACC) of these three models was 0.89, 0.84, and 0.84, respectively. The area under the subject curve (AUC) of these three models was 0.86, 0.92, and 0.93, respectively. As for Slope, TTP, and R parameters, the prediction ACC was 0.91, 0.89, and 0.81, respectively, while the AUC was 0.87, 0.85, and 0.83, respectively. In both the training and testing sets, the ACC and AUC of the combined model were higher than those of the radiomics models (ACC = 0.91 and AUC = 0.95), which indicate an outstanding performance of our proposed model.

**Conclusions:**

The radiomics nomogram demonstrates satisfactory predictive results for the treatment response of patients with osteosarcoma before NAC. This finding may provide a new decision basis to improve the treatment plan.

## 1 Introduction

Osteosarcoma is the most common primary malignant bone tumor, accounting for approximately 12% of primary bone tumors and mostly occurring in adolescents with a high degree of malignancy. However, a natural prognosis of osteosarcoma is extremely difficult. In the past, the 5-year survival rate of patients undergoing surgery alone was only 20–30% (1), and its diagnosis, treatment, and prognosis have been the research focus. With neoadjuvant chemotherapy (NAC), the 5-year survival rate improved to 60–80%, and the overall limb salvage rate increased from 10–20 to 80–90% (2). NAC is the most critical prognostic factor for osteosarcoma; except for operation, it can significantly extend the progression-free survival and improve life quality (3). The response to NAC has a direct influence on the formulation of the clinical treatment protocols. Therefore, effective evaluation of the efficacy of NAC is critical (4).

Currently, tumor necrosis rate after chemotherapy, calculated using postoperative pathological sampling, is used as the “gold standard” for evaluating NAC response. However, this method was invasive and involves a complicated operation. Random sampling or biopsy only examines part of the tumor tissues obtained, which cannot comprehensively assess the intra-tumor heterogeneity before operation. Therefore, it cannot be used for real-time monitoring or application of non-operative patients. As a non-invasive alternative, medical imaging has shown great potential in osteosarcoma (OS) diagnosis and in NAC efficacy evaluation. For the efficacy evaluation of NAC for osteosarcoma, previous reports mainly focused on routine scanning to determine tumor volume changes, diffusion-weighted imaging (DWI) signal characteristics, and MRI dynamic enhancement mode (5). However, tumor size does not reflect biochemical information within the tumor. In addition, DWI imaging presents large artifacts because osteosarcoma is primary to the bone and often accompanies tumor bone formation. The dynamic contrast-enhanced MRI (DCE-MRI) parameters are easily affected by arterial input function, model selection, individual cardiac output, blood pressure, and other factors. Such traditional imaging methods are insufficient to predict how well different patients will respond to NAC. In addition, due to the limitations of subjective factors such as the experience and knowledge of clinicians, the efficacy of NAC for osteosarcoma as evaluated by conventional MRI is still unsatisfactory. So far, no widely accepted clinical and imaging standards were constructed to evaluate the efficacy of NAC prior to medication to personalize a medication regimen.

More recently, with the advent of “radiomics”, there has been a growing focus on the discovery and usage of quantitative radiomics features of MRI images. Radiomics builds a relevant statistical model from a large number of high-dimensional extractable features from medical imaging data (possibly combined with clinical or genomic data) to assist in diagnosis, prognosis, and therapy monitoring. Machine learning is an important step in radiomics that involves building data-based computational models and methods to improve the accuracy, performance, or predictive power of the model. As a result, machine learning strategies have a strong prognostic and predictive performance, as well as excellent stability, which is required for radiomic-based analysis. In recent years, radiomics-based machine learning has been utilized in the diagnosis and prognostic assessment of liver, prostate, lung, and breast cancers (6, 7).

In particular, the radiomics characteristics of primary tumors have been proved to be closely related to how tumor responds to chemotherapy, and it has been reported that the radiological characteristics of primary colorectal cancer can successfully predict the efficacy of NAC (8), but the evaluation of NAC for osteosarcoma has rarely been reported.

In this study, machine learning-based radiomics nomogram was applied to identify poor histologic response to chemotherapy patients, which helps avoid ineffective multi-cycle chemotherapy in patients who do not respond well to chemotherapy. The aim is to provide guidance to clinicians with different therapeutic schemes, such as surgical tumor removal suggestion and chemotherapy regimen modification, to reduce the risk of disease progression and metastasis.

## 2 Materials and Methods

### 2.1 Patients and Dataset

This study was retrospective and has been approved by the institutional review board of our hospital, with informed consent of the patient being waived. A retrospective analysis of patients with osteosarcoma, as confirmed by surgery and pathology, between January 2016 and May 2020 was performed. The patient inclusion criteria were as follows: (1) osteosarcoma confirmed by histopathology, (2) MRI scans performed at two timepoints (within 1 week before the NAC implementation and at the end of the two cycles of NAC), and (3) more than two cycles of NAC treatment were performed in the local hospital. The exclusion criteria were as follows: (1) patients who did not undergo multiple-sequence MRI before or after NAC, (2) patients undergoing direct surgery without NAC, (3) patients who failed to complete the two cycles of NAC or discontinued the treatment, and (4) patients that were unable to provide complete MRI data. The specific diagnosis and treatment process of all patients were conducted under the consensus of experts of Clinical Diagnosis and Treatment of Typical Osteosarcoma (9). The specific NAC drugs are adriamycin (ADM)—60 mg/m^2^, cisplatin (DDP)—100 mg/m^2^, methotrexate (MTX)—10–12 g/m^2^, and ifosfamide (IFO)—10 g/m^2^. A chemotherapy cycle includes four rounds: the first ADM (D1–D3) and DDP (D4), the second MTX (D1), the third MTX (D1), and the fourth IFO (D1–D5). The disease development of patients should be observed after each round, and the effect of NAC is generally evaluated after the end of one chemotherapy cycle.

**Figure 1:**
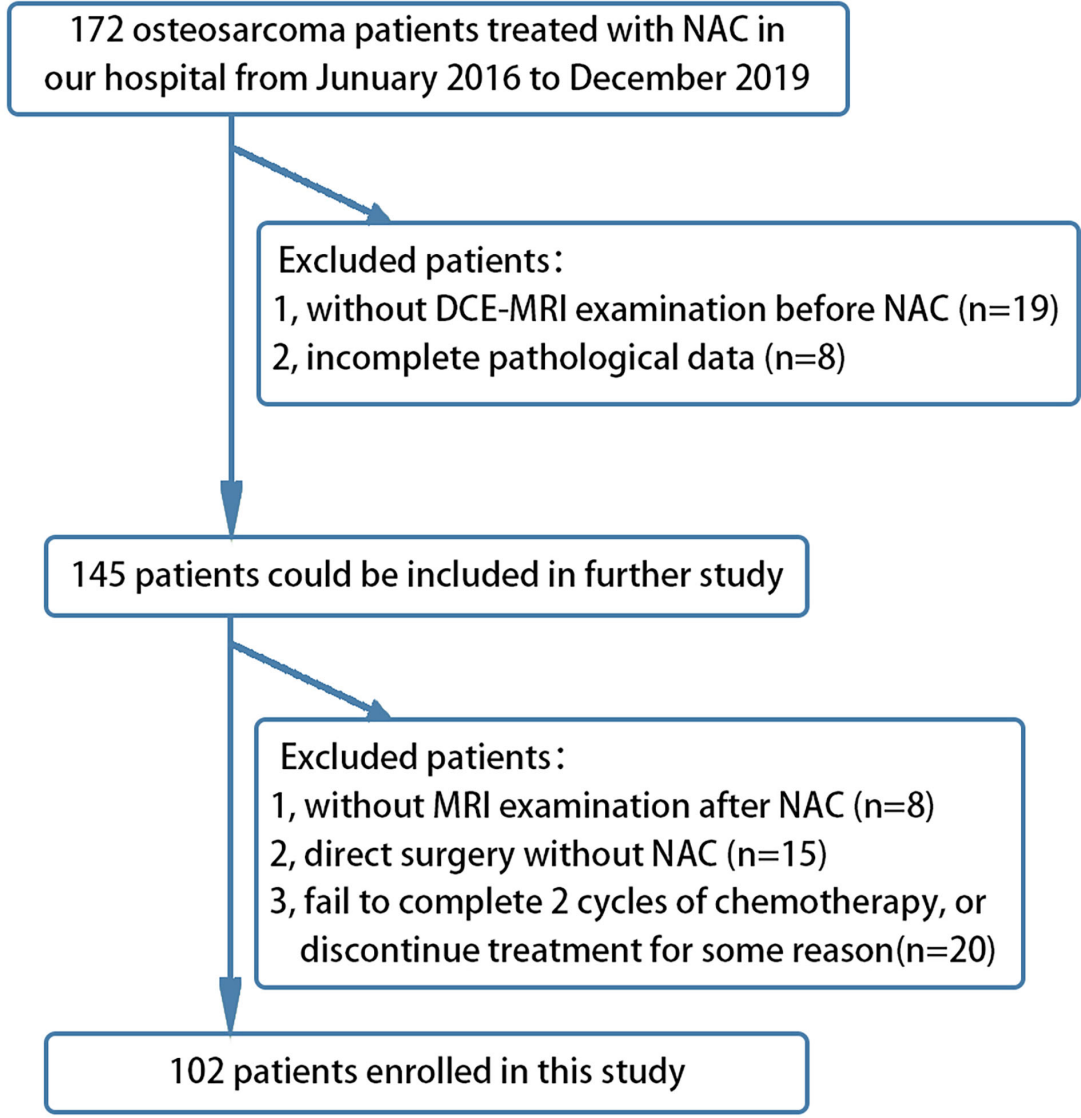
Flow chart showing the inclusion and exclusion criteria of this retrospective study.

A total of 172 patients with osteosarcoma who underwent NAC and surgical treatment were collected, and 35 patients with imperfect MRI and pathological data were excluded. Twenty patients had direct surgery but had not undergone NAC treatment; 15 patients failed to complete two cycles of chemotherapy or terminated chemotherapy. As a result, a total of 102 patients were included in the study (**Figure 1**). Two radiologists with 10 years of experience in musculoskeletal MRI diagnosis evaluated the tumor tissue independently (comparing images at the end of the first week of NAC and the MRI examination before NAC). The main evaluation focus was the comprehensive judgment of the changes in the size and shape of the lesion as well as the signal changes in each sequence before and after NAC. The efficacy of osteosarcoma treatment after two NAC cycles was evaluated according to RECIST (10), where complete remission (CR) was defined as no residual tumor, partial remission (PR) is defined as whether the longest diameter of the tumor is less than 70% of the original size, and progressive disease (PD) was defined if the total diameter of the target lesion increases by ≥20% or if new lesions appear. If the tumor change does not reach PR or PD state, it is defined as stable disease (SD). According to the abovementioned evaluation criteria, the patients were divided into (1) effective group (including CR and PR) and (2) ineffective group (including PD and SD). Intra-class correlation coefficient (ICC) was used to evaluate the consistency of the evaluation results between the two physicians.

### 2.2 Clinical and Pathological Data

Clinical information was collected from the medical record data, including age, tumor size, tumor site, pathological fracture, and surgical stage. Surgical staging was performed according to the surgical staging criteria for osteosarcoma (11).

### 2.3 MRI Examination and Image Analysis

All MRI scans were acquired at a local hospital on a Siemens Skyra 3.0 T MR scanner with (1) axial fat-suppressed T2WI: TR = 4,200 ms, TE = 100 ms, FOV = 260 mm × 350 mm, flip angle = 15°, slice thickness = 4.0 mm; (2) axial T1WI: TR = 500 ms, TE = 20 ms, FOV = 260 mm × 350 mm, flip angle = 15°, slice thickness = 4.0 mm; and (3) coronal fat-suppressed T2WI: TR = 4,200 ms, TE = 10 ms, FOV = 360 mm × 380 mm, flip angle = 15°, slice thickness = 4.0mm. Joint coil or body coil was selected based on the range and location of the tumor.

#### 2.3.1 DCE-MRI

First of all, a fat-suppressed T1WI (TR = 3.9 ms, TE = 1.3 ms, flip angle = 15°, FOV = 340 mm × 340 mm, slice thickness = 3.0 mm) scan was acquired before injecting the contrast agent. Then, 0.1 mmol/kg of body weight of the gadolinium-based agent (Magnevist; Bayer Healthcare, Berlin, Germany) was injected using a Medrad high-pressure syringe (rate = 2 ml/s), followed by injecting 20 ml normal saline into the tube. DCE images were acquired as six post-injection scans during the intravenous injection of Magnevist. The dynamic scanning time was approximately 360 s, with a temporal resolution of 3s.

#### 2.3.2 Conventional Semi-quantitative Parameters of DCE-MRI

The scanned images are fed into the post-processing workstation and processed through GE AW Volume Share 4.0. Regions of interests (ROIs) were manually delineated after having been discussed by two senior-level radiologists in musculoskeletal MRI diagnosis. The area of the ROI was about 10–20 mm^2^. The ROI was obtained from the layer with the best enhancement, avoiding necrotic tissue and blood vessels. Time–intensity curve (TIC) was automatically generated by the software. A gradual increase in the signal of solid tissue, without a well-defined shoulder, was defined as “curve type I”. A moderate initial rise in the signal, followed by a plateau, was defined as “curve type II”. An initial rise rapidly to a peak in the signal, followed by a decline, was defined as “curve type III”. Conventional semi-quantitative parameters of enhancement wash-in slope (Slope), time to peak (TTP), and enhancement rate (R) were obtained by calculating the TIC curve.

### 2.4 Radiomics Analysis

**Figure 2:**
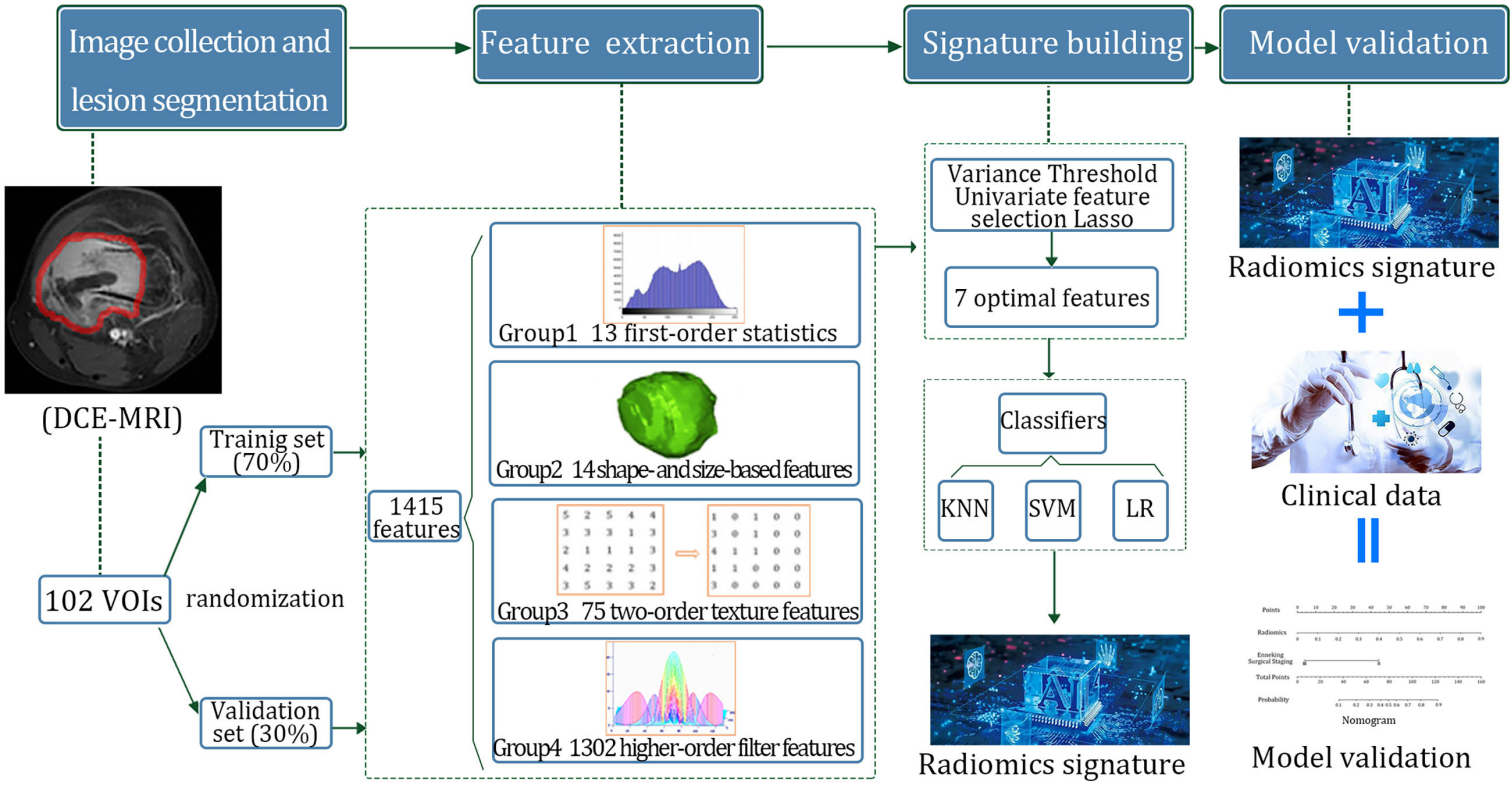
Flow chart of radiomics analysis and model construction. DCE-MRI, dynamic contrast-enhanced magnetic resonance imaging; VOI, volume of interest.

DCE-MRI sequence was used for radiomics analysis. The radiomics analysis method includes the following steps: image collection and lesion segmentation, feature extraction, feature selection, model construction, and prediction evaluation of the models (**Figure 2**).

#### 2.4.1 Image Normalization

To minimize MRI intensity variations, the image was normalized by centering at the mean with standard deviation using the following formula:


$$f(x)=\frac{s(x-\mu_{x})}{\sigma_{x}}$$

where *x* indicates the original intensity, *f*(*x*) indicates the normalized intensity, *μ_x_* refers to the mean value of the image intensity, *σ_x_* indicates the standard deviation of the image intensity, and s represents optional scaling, set to 1 by default. Normalization is based on the intensity of the entire image instead of just within the segmentation region.

#### 2.4.2 Image Segmentation

**Figure 3:**
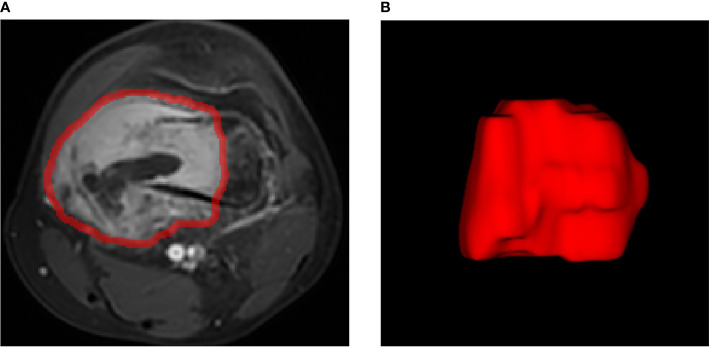
Example image for osteosarcoma contouring. **(A)** Outline of regions of interest on one slice of axial T1-weighted MR image of the strongest enhanced phase. **(B)** Volume rendering.

The volume of interest (VOI) was accurately delineated on the strongest enhanced phase using ITK-SNAP (version 3.8.0; http://www.itksnap.org). All the lesions (VOI) were manually delineated by one radiologist. The radiologist has 6 years of experience in musculoskeletal imaging diagnosis and was blinded to the clinical information of the patient. All delineations are then reviewed by a senior radiologist, and discrepancies are corrected based on tumor borders. The VOI should cover the entire lesion area, including the bone of the lesion, with its surrounding soft tissue mass, as well as the cystic necrosis area inside the lesion. The edema area and blood vessels around the lesion should be excluded. In this study, 102 VOIs delineated on scans from 102 patients were used for subject analysis (**Figure 3**).

#### 2.4.3 Radiomic Feature Extraction

A total of 1,409 quantitative imaging features were extracted from MR images with the Radcloud platform (http://radcloud.cn/). These features were divided into four groups. Group 1 (first-order statistics) consists of 18 descriptors that quantitatively describe the distribution of voxel intensities within the MR image through commonly used basic metrics. Group 2 (shape- and size-based features) contains 14 three-dimensional features that reflect the shape and size of the region. Group 3 (second-order texture features) contains 75 texture features that quantify region heterogeneity differences, calculated from gray-level run length and gray-level co-occurrence texture matrices. Group 4 (higher-order filter features) contains 1,302 first-order statistics and texture features after applying Laplacian, logarithmic, exponential, and wavelet filters on the image.

#### 2.4.4 Feature Qualification

The dataset was randomly separated into 70% for training and 30% for testing. To ensure the reproducibility of the extracted radiomics features, a feature consistency test of inter- and intra-raters was adopted. The evaluation was performed by randomly selecting 30 patients from the study and re-mapping the VOI using the same delineation protocol by the same radiologist and one other radiologist at 1 month after the initial delineation. Then, the ICCs of the quantized features extracted from the three VOI were calculated to evaluate the inter- and intra-rater consistency of image feature extraction, and features of ICC >0.85 were selected. Furthermore, feature selection methods, including variance threshold, SelectKBest, and least absolute shrinkage and selection operator (LASSO), were used to reduce redundant features. For variance threshold, features with eigenvalues of the variance smaller than 0.8 were removed. The SelectKBest belongs to a univariant feature selection method, and *p*-value was used to analyze the relationship between the features and the classification results. Features with *p*-value smaller than 0.05 were selected. LASSO filters variables and reduces model complexity. Variable screening refers to selectively putting variables into the model for fitting to get better performance parameters.

#### 2.4.5 Construction of Optimal Radiomics Signature Based on Machine Learning

Based on the selected features, three supervised classifiers were constructed for the radiomics-based models in this study: K-nearest neighbor, KNN; support vector machine, SVM; and logistic regression, LR. KNN calculates the distance between each sample point and all other sample points, keeps the k nearest neighbor points of the sample point, and assigns a class to the sample point by majority vote of the class of its k nearest neighbors. SVM combined the precision and function complexity of a given data to find the best proportion and to obtain the best generalization ability. LR includes classification, function establishment, solving optimal model parameters through optimization iteration, and verifying the model performance. To train the model, Gridsearch algorithm (Python scikit-learn library) was used for parameters that optimize the performance.

#### 2.4.6 Construction of Radiomics Nomogram

To test whether the combined clinical indicators and radiomics signatures improve the predictive performance, a multivariate logistic regression model was used to integrate the established radiomics labels and the clinical indicators in the training set to build a predictive model for the combined efficacy. Stepwise regression analysis and Akaikes information criterion (AIC) test are used to determine the best clinical information to be included from the candidate clinical indicators (age, maximum diameter of the tumor before treatment, tumor location, whether the tumor was associated with a pathological fracture, and surgical stage). A low AIC score indicates that the statistical model takes both complexity and accuracy into consideration. The model with the lowest AIC score provides the most effective independent factors, which optimizes the construction of a multi-factor regression model with the least parameters while avoiding over-fitting and ensuring desirable data fitting. Based on the combined model, the nomogram diagram transforms it into a visual therapeutic prediction model, providing a practical tool to predict the likely probability of individual chemotherapy effectiveness. The nomogram sets the corresponding score value according to the regression coefficient of each variable in the multivariate logistic regression equation (the contribution of each influencing factor to the efficacy) and then sums the score of each influencing factor to calculate the total score. The conversion function between the total score and the efficacy of chemotherapy (endpoint event) was then used to obtain the prediction probability of efficacy for each patient.

### 2.5 Statistical Analysis

We used Radcloud (Huiying Medical Technology Co., Ltd.) to manage imaging data, clinical data, and subsequent radiomics statistics analysis. Another statistical analysis was performed with SPSS 20.0 and MedCalc15.2.2. Differences of onset age and tumor size between the effective group and the ineffective group were compared by means of independent-sample *t*-test (two-tailed, *p* < 0.05). Mann–Whitney *U*-test was applied to compare the difference in tumor surgical staging, tumor site, pathological fracture, and the semi-quantitative parameters (Slope, TTP, R), with *P <*0.05 defining the difference as statistically significant. To assess the predictive performance of the classifiers, the receiver operating characteristic (ROC) curve, namely, area under the curve (AUC), was used in both training and testing datasets. Four indicators, including P [precision = true positives/(true positives+ false positives)], R [recall = true positives/(true positives+ false negatives)], f1-score [f1-score = P*R*2/(P + R)], and support (total number in test set), were selected to evaluate the performance of the classifiers in this study.

## 3 Results

### 3.1 Clinical and Pathological Features

There was a total of 102 patients with osteosarcoma (60 males and 42 females; mean age: 17 ± 9.77 years; range: 5–57 years), where 71 cases belong to the effective group and 31 cases belong to the ineffective group. Most clinical risk factors of the patients, including age, tumor size, pathological fracture, and pathological type, show no statistically significant difference between the effective group and the ineffective group (*P* > 0.05), except for surgical staging (*P* = 0.013). The effective or ineffective rate of chemotherapy was consistent between the training dataset and the testing dataset, and no significant difference was found in age, longest diameter of tumor, tumor location, and pathological fracture. The ratio of patients in each surgical stage was similar between the two sets (**Table 1**).

**Table 1:** Clinical features.

Clinical features	Training set			Test set		
	Effective group	Ineffective group	*P*-value	Effective group	Ineffective group	*P*-value
Age (mean ± SD)	18.49 ± 9.871	14.73 ± 9.331	0.171	17.14 ± 8.326	13.65 ± 8.141	0.243
Tumor size (mean ± SD) (cm)	11.79 ± 4.721	9.1550 ± 4.06873	0.406	12.82 ± 4. 324	10.01 ± 3.987	0.591
Tumor site Thigh bone Tibia Humerus	36 (35.29%) 16 (15.69%) 7 (6.86%)	15 (14.71%) 5 (4.90%) 2 (1.96%)	0.315	9 (8.82%) 2 (1.96%) 1 (0.98%)	6 (5.88%) 2 (1.96%) 1 (0.98%)	0.478
With pathological fracture	21 (20.59%)	13 (12.75%)	0.734	6 (5.88%)	3 (2.94%)	0.565
Surgical stage			0.013			0.025
II	47 (46.08%)	12 (11.76%)		9 (8.82%)	5 (4.90%)	
III	12 (11.76%)	10 (9.80%)		3 (2.94%)	4 (3.92%)	

### 3.2 Predictive Performance of Conventional Semi-quantitative Parameters of DCE-MRI

**Figure 4:**
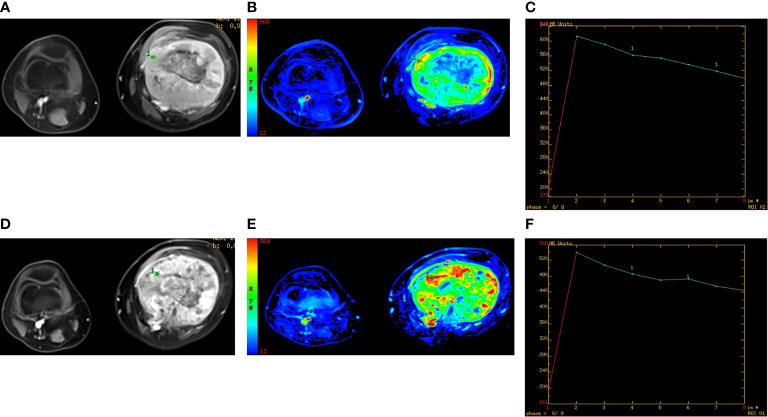
Male, 20 years old, left lower femoral osteosarcoma, ineffective group. **(A, D)** The dynamic enhanced axial T1WI fat suppression images before and after neoadjuvant chemotherapy (NAC). **(B, E)** The wash-in slope (Slope) images before and after NAC. The slope value and tumor active area increased. **(C, F)** The enhancement signal curve corresponding to the most obvious enhancement area, both curve type III before and after NAC.

After NAC, the Slope and R of the effective group decreased, and the TTP increased. The difference of each semi-quantitative parameter before and after NAC was statistically significant (*P* = 0.013). There is no statistically significant difference in Slope and R (*P* = 0.350) before NAC; while after NAC, the differences in Slope, TTP, and R between the two groups becomes statistically significant (*P* = 0.023). There was a difference in TIC distribution before and after NAC in the effective group (*P* = 0.002). There was no significant difference in TIC distribution before and after NAC in the effective group (*P* = 0.570) (**Figure 4**). There was no significant difference in TIC distribution between the two groups before chemotherapy (*P* = 0.103). The ROC curve was used to predict the importance of each semi-quantitative parameter on the efficacy of NAC in osteosarcoma. Taking the RECIST standard as the curative effect classification standard, the sensitivity and specificity of the three parameters (Slope, TTP, and R) to predict a desirable response to osteosarcoma after chemotherapy were 0.83, 0.92, and 0.91 and 0.69, 0.85, and 0.75, respectively. The AUC was 0.87, 0.85, and 0.83, respectively.

### 3.3 Radiomic Features and Predictive Performance of the Classifiers

**Figure 5:**
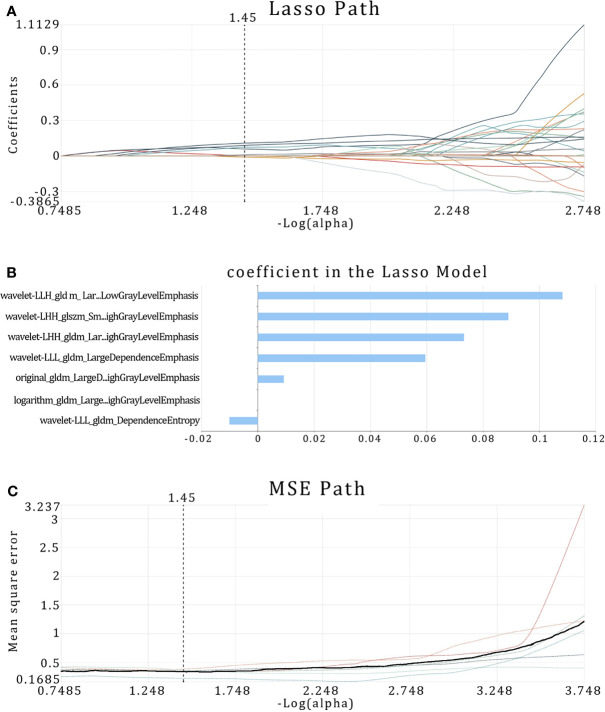
Radiomic feature selection using least absolute shrinkage and selection operator (LASSO) regression. **(A)** MSE path, the black solid line is the mean value of mean square error, and the maximum number of iterations is 100. Ten-fold cross-test was used to find the optimal -log(alpha) value of 1.45. **(B)** LASSO path, the radiomic features change with alpha value. **(C)** Histogram showing the selected seven features and their coefficients in LASSO model. Using LASSO model, seven features which correspond to the optimal alpha value were selected.

In the first step, the reproducibility ICC score of each feature was used to evaluate feature stability, and features with ICC >0.85 were selected. According to this criterion, 863 (61.2%) stable features were reserved for further screening. Then, we selected 446 features from 863 features using the variance threshold method. Furthermore, 74 features are selected with the select K best method. Finally, LASSO regression was performed to select 74 radiomics features for dimensionality reduction and 10-fold cross-test to obtain an optimal -log(alpha) value of 1.45 (**Figure 5**). According to the alpha value, we find the coefficient of different features, then select the features with non-zero coefficient, and finally obtain the most relevant features. Finally, seven radiomics features with the best correlation with NAC curative effect are selected to construct prediction models. These seven features were all texture features, and five of those with the highest LASSO regression coefficient were features after wavelet transform. The selected features and their corresponding regression coefficients, associated feature groups, and filters are shown in **Table 2**.

**Table 2:** Description of the selected radiomic features with their associated feature group and filter.

Filter	Radiomic class	Radiomic feature	Coefficient
Wavelet-LLL	GLDM	Large-dependence emphasis	0.05951
Wavelet-LHH	GLDM	Large-dependence high-gray-level emphasis	0.07326
Wavelet-LLL	GLDM	Dependence entropy	-0.01009
Original	GLDM	Large-dependence high-gray-level emphasis	0.00924
Logarithm	GLDM	Large-dependence high-gray-level emphasis	0.00001
Wavelet-LLH	GLDM	Large-dependence low-gray-level emphasis	0.10825
Wavelet-LHH	GLSZM	Small area high-gray-level emphasis	0.08899

GLDM, gray-level dependence matrix; GLSZM, gray-level size zone matrix.

**Figure 6:**
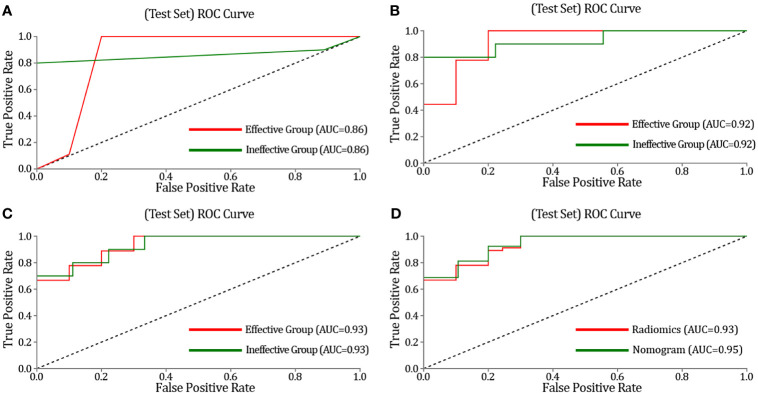
The receiver operating characteristics (ROC) curves of the K-nearest neighbor (KNN), support vector machine (SVM), logistic regression (LR), and the nomogram in the test. **(A)** ROC curves of KNN methods for classification. **(B)** ROC curves of SVM methods for classification. **(C)** ROC curves of LR methods for classification. **(D)** Prediction performance of the ROC curves for the nomogram and radiomics signature (the model of LR). The nomogram provides higher area under the subject curve values than the radiomics signature.

The three classifier models (KNN, SVM, and LR) are separately built by learning feature values extracted from DCE-MRI to evaluate the training set and the testing set of the model. The performance of KNN, SVM, and LR classifiers are compared *via* AUC, sensitivity, and specificity (**Table 3**). These classifiers exhibit a satisfying performance of NAC for OS classification on the ROC curve, with an AUC of 0.86, 0.92, and 0.93, respectively (**Figure 6**). The AUC of the SVM and the LR classifier is the most optimal. When the SVM classifier is used for training, the sensitivity and specificity of the effective group are 0.82 and 0.85, and these were 0.85 and 0.82 for the ineffective group, respectively. The 95% CI is 0.85–1.00, and the accuracy of the training set and the testing set was 0.89 and 0.84, respectively. When the SVM classifier was used for training, its accuracy, recall rate, and F1 value were 0.78, 0.78, and 0.78 in the effective group, respectively, and 0.80, 0.80, and 080 in the ineffective group, respectively. At the same time, when the LR classifier was used for training, the results were also optimal. The sensitivity and specificity of the effective group were 0.85 and 0.85, respectively. The sensitivity and specificity of the ineffective group were 0.85 and 0.85, respectively. The 95% CI was 0.81–0.98, and the accuracy of the training set and the testing set is 0.86 and 0.84, respectively. When using the LR classifier for training, its accuracy, recall rate, and F1 value were 0.80, 0.89, and 0.84 in the effective group, respectively, and 0.89, 0.80, and 084 in the ineffective group, respectively. Such results indicate that the model, based on radiomics features, has adequate efficacy differentiation ability in both the training set and the testing set.

**Table 3:** Receiver operating characteristics and three indicators -Precision, Recall, F1-score results with K-nearest neighbor (KNN), support vector machine (SVM), and logistic regression (LR) classifiers of test set.

Classifiers	Efficacy	AUC	95% CI	Sensitivity	Specificity	Precision	Recall	F1-score
KNN	Ineffective	0.86	0.74–0.99	0.8	1	1	0.8	0.89
	Effective	0.86	0.74–0.99	1	0.8	0.82	1	0.9
SVM	Ineffective	0.92	0.72–1.00	0.8	0.78	0.8	0.8	0.8
	Effective	0.92	0.72–1.00	0.78	0.8	0.78	0.78	0.78
LR	Ineffective	0.93	0.75–1.00	0.8	0.89	0.89	0.8	0.84
	Effective	0.93	0.75–1.00	0.89	0.8	0.8	0.89	0.84

### 3.4 Prediction Model of the Radiomics Nomogram

**Figure 7:**
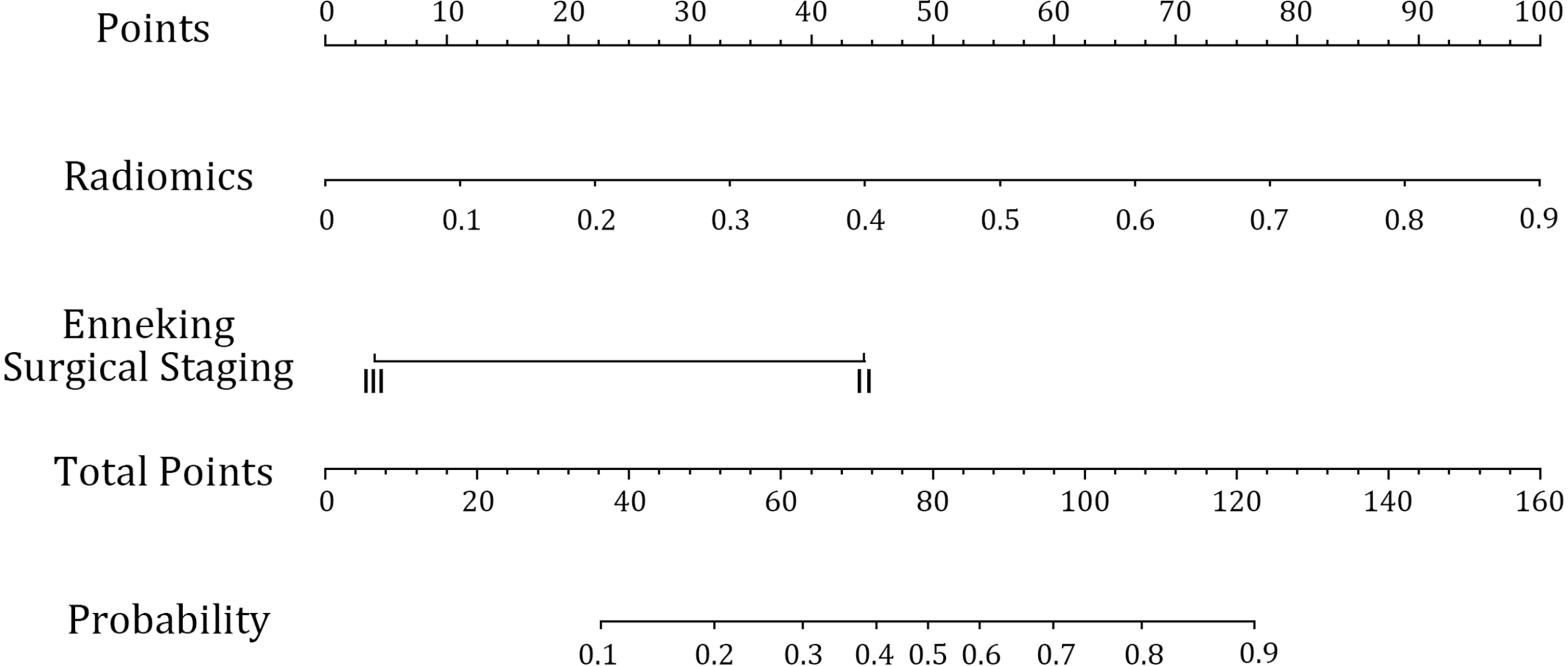
Radiomics nomogram to predict the efficacy of neoadjuvant chemotherapy. The radiomics nomogram is developed in the training set with radiomics signature and surgical staging.

After constructing the radiomics labels, the combined prediction model was formed by combining the labels with clinical indicators. After AIC stepwise regression, surgical staging was determined as the optimal clinical index. The combined model was successfully constructed using a multivariate logistic regression model to integrate surgical staging and radiomics labels and then demonstrated using a visual nomogram (**Figure 7**). The nomogram scores were given based on the weights of independent variables in the regression model, and the scale length of the nomogram variable is positively correlated with its impact on the efficacy prediction. Out of the two factors, the radiomics label contributed the most to predicting the outcome (the longest scale), followed by surgical staging. The high-probability segment of the radiomics label corresponds to the high-score area (score axis), and the low-probability segment corresponds to the low-score area. The probability of chemotherapy sensitivity in patients with earlier surgical staging is higher than that in patients with later staging. The scores of all factors were added up to obtain the total score, which was perpendicular to (probability axis of chemotherapy effect) obtain the probability of individual final chemotherapy effect. In addition, the integration of the surgical staging, prediction model of joint radiomics tag, presents desirable prediction performance. The training set classification accuracy is 0.91, while that of the testing set was 0.90, which are both higher than the performance of the simple radiomics group learning model. Furthermore, the AUC of the combined model was also significantly higher than that of the radiomics model, with the training set at 0.94 and the testing set at 0.95. It also reveals that the prediction efficiency of the radiomics nomogram was better than that of conventional semi-quantitative parameters (with AUC of 0.87, 0.85, and 0.83, respectively) of DCE-MRI. The AUC of the combined model was higher than that of the radiomics model in both the training set and the testing set. In conclusion, the combination of radiomics features and surgical staging improves the capability of curative effect prediction and improves the performance of the combined model with more accurate prediction results than the single radiomics model.

## 4 Discussion

Although the introduction of NAC in OS treatment has improved the survival rate of patients, local recurrence or metastasis still occurs in approximately 30 to 40% of the patients due to tumor heterogeneity, and there are significant differences in tumor biological behavior and response to NAC in different patients (12). In recent years, with the development of medical image analysis technologies in machine learning, the concept of “radiomics” has emerged. The quantitative image features extracted from conventional images are called “imaging features”, which include not only the intensity, shape, size, and volume information of the lesion but also the texture features and the high-level wavelet transform features which are difficult to observe with the naked eye (13). Unlike a needle biopsy that only partially provides pathological information of the tumor, radiomics features provide information in the three-dimensional volume of the tumor, which reflect the heterogeneity of the tumor and provide a comprehensive view of the tumor for treatment guidance. Furthermore, it is a non-invasive method that enables constant tumor change monitoring as well as constant tumor response to treatment evaluation (14, 15). In this study, we aim to establish the best artificial intelligence model based on radiomic features extracted from original MRI images of osteosarcoma patients. Thus, we can individually evaluate the efficacy of NAC.

Our research demonstrates that, besides surgical staging, other clinical characteristics, including age, tumor size, whether pathological fracture occurs, and tumor site, are not adequate as independent risk factors to predict the effectiveness of NAC for osteosarcoma, which is similar to the result of a previous study using traditional methods (16). For stage III patients who have already metastasized, poor efficacy indicates that earlier tumor detection results in better efficacy of NAC, which also agrees with previous research results (17). The insignificance of other clinical indicators may be due to an inadequate sample size, limited data source, and geographical constraints. In this study, the DCE-MRI sequence is selected for feature extraction because of thinner slice thickness and higher image resolution, which displays a more distinct tumor boundary and reflects more heterogeneous information such as blood supply inside the tumor (18). We did not include DWI sequence due to unclear lesion boundaries on DWI, poor image resolution, and difficulties to completely segment the lesions, especially the adjacent bone parts. Kickingereder et al. (19) used MR imaging radiomic features to predict the survival of bevacizumab in the treatment of recurrent glioblastoma and obtained more radiomic features from the enhanced T1WI sequence (62.5%) than from the T1WI and T2-FLAIR sequences (37.5%), and the prognostic weight of the radiomic features obtained from the enhanced sequence is also higher than that of the plain scan sequence. Therefore, compared with conventional MR sequence, image segmentation on the enhanced sequence produces more radiomic features, which is more valuable for model construction.

In this study, we analyze the data from 102 patients with osteosarcoma, and we used machine learning to validate that MR radiomics features before NAC therapy are closely related to tumor heterogeneity and the efficacy of NAC. Seven high-level statistical features were selected from the DCE-MRI images that highly relate to the NAC efficacy. Such features are indicators of strength and texture, which effectively reveal the heterogeneity and subtle changes in tissue morphology within the tumor (20, 21). Five of the features were texture features after wavelet transform. Wavelet transform calculates the resolution of the signals at different time, space, and frequency scale planes. Therefore, in various researches on radiomics, texture features after wavelet transform are used to construct prediction models. Hu et al. pointed out that, compared with gray-level co-occurrence matrix, wavelet features extracted from bone DR have higher accuracy in diagnosing osteosarcoma. Mahrooghy et al. found that wavelet features extracted based on DCE-MRI can effectively reflect the heterogeneity of breast cancer, and the constructed breast cancer prognosis classification model also has high predictive performance (22). Of these seven features, six features are gray-level dependence matrix features, and one is gray-level size zone matrix feature, indicating the importance of gray values in NAC efficacy evaluation. This assumption is consistent with the results of previous imaging studies on the prediction of sentinel lymph node metastasis in breast cancer (23). Among these features, the most important features with the highest LASSO coefficient are large-dependence low-gray-level emphasis and small-area high-gray-level emphasis. They measure the joint distribution of large dependence with lower gray-level values ​​and the proportion in the image of the joint distribution of smaller-sized zones with higher gray-level values, respectively. Furthermore, previous studies on cardiac SPECT radiomic feature also emphasized the importance of these two features (24).

We aim to establish the best artificial intelligence model to predict the efficacy of NAC for osteosarcoma treatment. In our study, SVM and LR performs better than KNN, possibly because the manual delineation of lesions adopted in this study was affected by human factors. Osteosarcoma is often mixed with the formation of tumor bone, leading to the inclusion of undesired tumor bone components in manual segmentation. The feature parameters extracted from the images of these tissues are less capable of differentiating subtle differences between groups, resulting in the low diagnostic efficiency of KNN. LR is a classical classifier which provides not only the results but also the corresponding probabilities, so the fitted parameters can clearly display the impact of each feature on the result. However, it is essentially a linear classifier, which is not applicable when the correlation between features is high. It is also not robust to outliers. In this study, the SVM classifier also has a high predictive performance, with the sensitivity, specificity, and AUC of the testing set being 0.79, 0.79, and 0.93, respectively. Thus, the appropriate classifier should be selected based on the specific situation. LR is suitable for the case whose number of features is large and is similar to the number of samples. SVM is suitable for a small number of features with general samples that are neither too large nor too small. Our results suggest that a classifier based on radiomics features extracted from the best-enhanced DCE-MRI can be used as a new method to predict the efficacy of NAC. This method can help determine the efficacy of NAC before surgery. Therefore, it is capable of assisting clinicians to determine appropriate treatment plans for patients. Previously, several reports have demonstrated that predictive models based on radiomics features can be used to predict the efficacy of colorectal, lung, and liver cancers (6, 7, 25).

The semi-quantitative parameters of DCE-MRI effectively reveal the characteristics of tumor angiogenesis: the level of Slope and TTP represents the amount of new angiogenesis in tumor tissue, and R reflects the overall vascular density and permeability of the tumor. We adopt ROC curve analysis in this study and found that Slope, TTP, and R also exhibit notable predictive efficacy in predicting the NAC response of OS, and Slope receives the highest value. However, a nomogram based on radiomics offers better predictive performance. The reason may be that traditional semi-quantitative parameters are based only on imaging, but a nomogram combines a large amount of high-throughput imaging information and clinically valuable indicators to form a more comprehensive evaluation model. It customizes the treatment for patients more accurately and improves the prognosis. The combined model, nomogram, has high predictive accuracy and is convenient and practical, and it has broad clinical application prospects in the treatment of OS. This model is helpful to optimize the treatment strategy of locally advanced cervical cancer. Currently, there is a lack of effective markers to predict the efficacy of chemotherapy in clinical practice, and it is uncertain whether patients should receive preoperative NAC. A nomogram enables doctors to identify chemotherapy-sensitive or chemotherapy-insensitive patients before the initial treatment to develop appropriate regimens. For insensitive patients with large tumor foci, simultaneous radiotherapy and chemotherapy should be selected immediately, and for young patients with chemotherapy-sensitive patients, NAC combined surgery is recommended. In addition, due to the deficiency of radiotherapy facilities in some developing countries, NAC has been explored as an advanced treatment for tumor control before concurrent radiotherapy and chemotherapy. The combined radiomics model can also assist in identifying patients who are suitable for NAC prior to concurrent radiotherapy and chemotherapy. Furthermore, the nomogram combined model may be able to predict the response of patients with recurrent or metastatic cervical cancer to systemic chemotherapy using preoperative MRI images. If the nomogram combined radiomics model can be utilized in clinical practice, it will be beneficial to promote personalized precision medicine for OS.

In conclusion, our study demonstrates the feasibility of combining artificial intelligence with MRI radiomics to predict the efficacy of NAC for osteosarcoma by depicting VOI during the most intense period of enhancement. This is a noninvasive and highly accurate method that can predict the efficacy of NAC for osteosarcoma before surgery. It helps avoid ineffective multi-cycle chemotherapy in patients who do not respond well to chemotherapy as well as guide the clinical doctors with a different therapeutic schedule.

### 4.1 Deficiencies and Prospects of This Study

Due to sample size limitation, the number of cases used in the testing set of this study is small. It is necessary to further increase the sample size of the testing set in the future to compare the effectiveness and practicability of these classifiers. Furthermore, this study only used radiomics and clinical features to construct the NAC efficacy prediction model, without the pathological information of the patients. Further research is needed to construct a more comprehensive prediction model combining clinical, pathological, and imaging features. In addition, VOI in this study is manually delineated. Since the delineation process is time-consuming, this study adopted the sketching scheme of the review of young doctors and senior doctors, without repeated sketching of lesion areas. In subsequent studies, the accuracy and repeatability of VOI should be further evaluated. For research with a larger sample size in the future, semi-automatic or automatic segmentation can be adopted to reduce time cost.

## Data Availability Statement

The original contributions presented in the study are included in the article/**Supplementary Material**. Further inquiries can be directed to the corresponding author.

## Ethics Statement

This study was retrospective and was approved by the Institutional Review Board of People’s Hospital of Zhengzhou University, and informed consent of the patients was waived.

## Author Contributions

YG was the general responsible person of the project, responsible for the design of the project, writing and checking the article, and responsible for the reliability of the article. YG also formulated the overarching research goals and aims. HL participated in the development of the whole experiment, planned the main parts of the experiment, and analyzed the results. HL was also responsible for the detailed analysis and delineation of patients’ MRI images. YG and HL are also responsible for the coordination and review of this study. TC participated in the planning and execution of the experiment, and assisted in the specific analysis, delineation, and verification of patients’ MRI images. LZ was responsible for the design of the experiment, the exploration and implementation of the radiomics methodology, the analysis of the results, and the writing of the main part of the manuscript. FZ was responsible for the collection, sorting, classification, and statistics of clinical information of patients. YX was responsible for software design, computer programming, and data analysis related to the radiomics portion of the study. QG was responsible for patient enrollment, imaging and clinical information collection, and data collation. All authors contributed to the article and approved the submitted version.

## Conflict of Interest

YX was employed by the company Huiying Medical Technology Co., Ltd.

The remaining authors declare that the research was conducted in the absence of any commercial or financial relationships that could be construed as a potential conflict of interest.

## Publisher’s Note

All claims expressed in this article are solely those of the authors and do not necessarily represent those of their affiliated organizations, or those of the publisher, the editors and the reviewers. Any product that may be evaluated in this article, or claim that may be made by its manufacturer, is not guaranteed or endorsed by the publisher.
